# Alcohol consumption and associated factors among undergraduate regular students in Wolaita Sodo University, Southern Ethiopia, 2021: a cross-sectional study

**DOI:** 10.11604/pamj.2023.45.179.35980

**Published:** 2023-08-24

**Authors:** Getahun Boltana, Mesfin Markos Kacharo, Amene Abebe, Daniel Baza

**Affiliations:** 1 Department of Boloso Sore Health Office, Wolaita Zone, Southern Ethiopia, Ethiopia,; 2 Department of Midwifery College of Medicine and Health Science, Wolaita Sodo University, Wolaita Sodo, Ethiopia,; 3 Department of Reproductive Health and Human Nutrition, Wolaita Sodo University, Wolaita Sodo, Ethiopia,; 4 Department of Pediatrics and Neonatal Nursing, Wolaita Sodo University, Wolaita Sodo, Ethiopia

**Keywords:** Alcohol consumption, risk factors, undergraduate graduating university students

## Abstract

Introduction: alcohol consumption is a major of public health problem in the worldwide. It has been linked to risk of nutritional related chronic diseases and one of the most common risks taking behaviors among young population in University students. Objectives: aimed to assess the prevalence of alcohol consumption and associated factors among undergraduate graduating regular students in Wolaita Sodo University.

Methods: a cross-sectional study design was conducted at Woliata Sodo University among undergraduate graduating regular students. Structured, self- administered questioner used to collect data by multistage sampling technique. Data were entered, cleaned and analyzed by using SPSS version 24. Multivariate logistic regression analysis used to decide variables with p<0.05**) as statistically significant**.

Results: four hundred and forty-six (446 (60.7%) of respondents consumes alcohol out of 735 respondents. The associated factors for of alcohol consumption were being female respondents AOR 0.34 95% CI: (0.21-.54), family history members consumes alcohol 4.8 times (AOR= 4.83, 95% CI: (2.68-8.70), who don´t know well about the effect of consuming alcohol were around 2.7 times (AOR= 2.71, 95% CI:( 1.67-4.50) being drunker friend were (AOR=0.03, 95% CI: 0.02-0.06), being chew “chat” use (AOR=0.45, 95% CI: (0.32-0.63), being smoking cigarettes use (AOR= 0.49, 95% CI: 0.29-0.88) were found to be significantly asso**ciated**.

Conclusion: the prevalence of alcohol consumption was relatively high compared to previous study. Attention should be given to counseling and peer education training and Anti-psychoactive substance club and sensitization therapy that are designed to change students´ perceptions on alcohol consumption.

## Introduction

Alcohol consumption is an important risk factor for many socioeconomic and health problems in many societies which is especially affects young common among the University students. It’s one of the most significant risks taking manners among adolescents and young population in University students are at a particular stage to experience more freedom in making personal choices about their health behaviors´ than previous or currently in life of health hazardous such as alcohol consumption peak in this age group [1,2]. According to the alcohol consumption study, drinking guides provide an indication of alcohol usage and can be used to calculate the contribution to the associated illness burden. It discovered three drinking patterns: abstention, occasions, moderate drinking scores, and excessive episodic drinking. The American Dietary Guidelines for 2015-2020 do not recommend that people begin drinking alcohol for any purpose [3]. It also causes numerous health-related habits to shift, triggering the onset or reinforcement of major non-communicable diseases during this phase of life. These behaviors have a negative impact on today's children's health and development, as well as a negative impact on their health, such as overweight and obesity [4]. Another study indicates that alcohol consumption is dramatically increasing among youth in Ethiopia, factors such as freedom from family control, psychological stressors related to the demand to adapt to new environment, academia, and makes new friends in university students at greater risk of alcohol consumption that lead to a wide range of societal problems, nutritional related chronic diseases and absent class in the student population [5]. College students have been reported to consume alcohol to potentially relax or relive tension, celebrate feel comfortable with the opposite sex as reward for working hard and to get way from difficulty and affect the behavior of University students this in turn is consuming alcohol followed by meal skips, decreased academic performance and many health problems [6]. Study show that many areas of a student´s life can be affected by alcohol consumption and alcoholism participate directly and indirectly in the development of physical, mental, malnutrition and socio-economic loss to individuals [7]. In college, young students are in a time of transitions and experimentation, sometimes including experimentation with alcohol and other drugs in many countries increase the risk of a wide range of health risks, public harms in the student population and alcohol consumption which is considered one of the leading pattern of today [8,9].

Alcohol consumption is a major public health problem issue in the worldwide. It is one of the most important risk behaviors among young University students. According to the Global Case Report from 2014, 26.5% of university students and adolescent age are current alcohol drinkers, resulting in 2.5 million people dying each year from alcohol-related causes and 320, 000 young people aged 15-29 years dying annually from alcohol-related causes, accounting for 9 percent of all deaths in substance abuse [10]. Alcohol intake is attributed to a number of health problems, including obesity, type 2 diabetes, hypertension, mental illness, and coronary heart disease [11]. According to another study, alcohol intake has a harmful impact on a number of medical conditions, including infectious diseases, cancer, neuropsychiatric disorders, cardiovascular diseases, pancreatic diseases, and unintentional and deliberate injuries [12]. Alcohol consumption is a huge societal burden, resulting in financial losses, health hazards, hunger, crime-related expenditures, and productivity losses [13]. In both the short and long term of impacts, alcohol consumption in university students was highly diverse and generally higher in developed nations in adolescence can raise the chance of developing neuron-cognitive disorders [14]. In Europe the prevalence alcohol use among University students was reported as follows general ranged from 53% in Ireland, 2.5% in Germany to 22% in Ethiopia [15]. It can also lead to eating disorders are common with the prevalence of 20% of women and 5% of men among college students [16]. Alcohol using students mostly females have been reported to the risk of the following four eating behaviors associated with drinking: skipping meals or self -regulating calorie intake during a meal to account for calories ingested from drinking altering drinking behaviors [17]. Its consumption lead to anorexia and bulimia occur in a relative percentage of women (0.5- 3%) a much higher percentage of young women report “subclinical” disordered eating behaviors [18]. In sub-Saharan Africa countries like Kenya ever drinking prevalence of alcohol were (22%) found among University students [19]. Where some study from South Africa also reported an alcohol use prevalence of 70% University students of adolescents [2]. Another study of Uganda has been reported to have the prevalence of alcohol consumption were 31% of University students in the East African region [20]. Another study of South Sudan in previous 30 days the prevalence of alcohol consumption 20.3% of University students [21]. Alcohol consumption of students in University has turn out to be intolerable not only that alcohol risk to health but also showed negative effects on the motivations for study and associated with poor academic performance, malnutrition, gastritis [22]. In Ethiopia studies indicated that the prevalence of alcohol use among college and University students was 26.65% [23]. Therefore, the aim of this studies to assess prevalence of alcohol consumption and associated factors among undergraduate graduating regular students of Wolaita Sodo University.

## Methods

**Study area and period:** the study was conducted at Wolaita Sodo University which is one of the public higher education institutions in Ethiopia, its located in Southern Nations Nationalities and People Region, Wolaita Sodo town around 330 km away from Addis Ababa via Alba. During the study period the University had six Colleges, five school and forty-two departments University teaching courses in regular, weekend, summer programs. This study includes undergraduate graduating regular students which are totally, 6000 (3594 males and 2406 female).

**Study design:** facility based cross-sectional study design was applied.

**Source population:** all undergraduate graduating regular students of Wolaita Sodo University in 2021 were taken as source population for this study.

**Study population:** the study populations were all undergraduate graduating regular students of different departments that randomly selected to participate in study.

**Inclusion criteria:** inclusion criteria of study were all undergraduate graduating regular students of 2021 who were randomly selected.

**Exclusion criteria:** weekend, summer, post graduate and PhD students were excluded from the study.

### Sample size

**1^st^ objective:** sample size was determined by using the single population proportion formula. It was calculated by an assumption of 67.6% respondents the prevalence of alcohol uses from previous study conducted in Dill University Southern Ethiopia availability [22]. The calculated sample size was 340. So with adjustment for non-response (10% contingency) n = (340+34) =374 and again multiplied by design effect two for multistage sampling the total sample size was 748 regular students.

**2^nd^ objective:** to determine sample size for associated factors, by using Epi info version 7 statistical programmers from different studies factors associated with alcohol consumption among undergraduate regular students with confidence interval 95%, power of the test 80% and margin of error 5%. We were used the first object of sample size because the second object sample size lessen the first object of sample size therefore the above sample size calculations the highest value was the final sample size for this study. So the final sample size for this study was 748.

**Figure 1:**
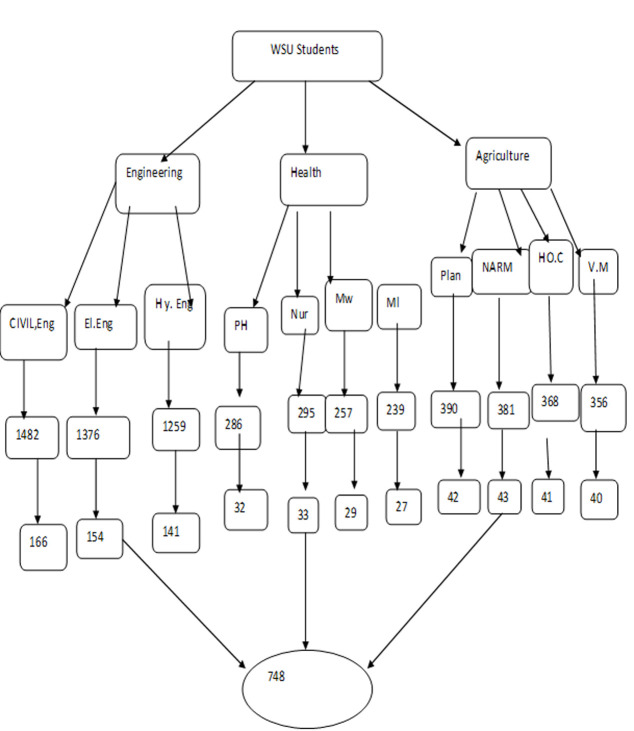
schematic presentation of sampling procedure, Wolaita Sodo University, 2021

**Sampling procedure** a multi-stage sampling procedure was applied to select sample of undergraduate graduating class students in the Wolaita Sodo University. Firstly, stratification was used to divide the students into graduating year of 3, 4 and graduating year of 5 and above of the three collages from the six collages of university these collages are College of engineering and technology, college of Medicine and Health sciences and college of agriculture selected randomly by simple random method again from 42 departments of these collages 11 departments were selected by simple random sampling. The calculated sample size was taken from proportionally allocated to each department based on the number of graduating students (Figure 1).

### Study variables

**Dependent variables:** alcohol consumption.

**Sociodemographic and economic:** sex, age, religion, year of study, occupation of family, income, family H x of alcohol use, and culture.

**Individual factors:** knowledge and attitude towards the effect of alcohol consumption.

**Peer pressure factors:** chewing chat, smoking cigarette, drunker friend and friends’ psychoactive substance, social class, academic performance.

**Environmental factors:** availability of alcohol beverage and access of information.

### Operational definitions

**Alcohol:** is an intoxicating substance, the active ingredient in drinks consumed and any locally available drink (beer, areqe, tella, teje and wine) which has stimulant effect.

**Alcohol consumption:** is a respondent who used alcohol at least once during the past 30 days before the survey [9].

**Audit:** is alcohol use disorder identification test to assess alcohol consumption survey was developed by World Health Organization as simple method of screening for alcohol consumption in the past 30 days.

**Good knowledge:** if the respondents have scored ≥70% about the consequence of alcohol consumption.

**Poor knowledge:** if the respondents have scored less than ≤60% about the consequence of alcohol consumption.

**Positive attitude:** a student who has a combined attitude value which merged from 5 attitudinal statements response was greater than or equal to the mean score value of all students.

**Negative attitude:** a student who has composite attitude value which merged from 5 attitudinal statements response was lower than mean score value of all students.

**Data collection procedure and tools:** data was collected by using structured self- administered questionnaire that adopted from reviewed literatures and studies conducted in University students. The data were collected by six-degree holder´s health professionals and supervised by two master holder public health professionals two days of training was provided to data collectors and supervisors. Alcohol consumption was measured by Alcohol used disorder identification test is a 10 item screening tool developed by World Health Organization (WHO) to assess alcohol consumption, drinking behavior and alcohol related problems that quantity of frequency retrospective estimate of average daily consumption. According to AUDIT scores for each question range from 1-7 indicates low risk, 8-15 indicates moderate risk,16-19 indicates high risk, 20 or more indicate possible dependence [9].

**Data quality assurance:** the data quality was assured by different methods of the questionnaire that was translated from English to Amharic by language expert to maintain its consistency. Two days of training was provided to data collectors and supervision the data collection tools and data collection procedures and the questionnaire validated by pre-test was conducted on 5% of sample size other Hossana University regular students outside study area. For daily activities data collectors were supervised closely by supervisors of the principal investigators and data collection process to ensure the completeness and consistency of gathered information. Then data was crosschecked and cleaned manually for completeness before entering in to Epi data and Statistical Package for Social Science (SPSS) and cleared if there is any mistake.

**Data analysis:** all data was checked for completeness and internal consistency by cross-checking then cleaned, edited, entering coded after it was entered in to Epi-Data version 3.1 and was exported to SPSS version 24 computer programs for further analysis and cleaning. Descriptive statistics was conducted to determine the frequencies, percentages and cross tabulations were done for the main variable to present prevalence of alcohol consumption experience by different factors considered or different variables and bivariate and multiple logistic regression analysis was done to identify variables which were associated with the dependent variables. Multivariate logistic regression analysis was carried out to examine the associations between each independent predictor and outcome variable. The model was checked for fitness with Hosmer and Lemeshew goodness of fit test was used to check the fitness of the model fitness. All variables with a p-value 0.25 in bivariate analysis were considered as the candidate for multivariate logistic regression to control possible confounders. Finally, variables with a p-value of <0.05 were having a statistically significant association with alcohol consumption at corresponding 95% CI.

The Wolaita Sodo University Institutional Review Board provided an ethical clearance letter and permission. All schools received a letter from Wolaita Sodo University College of Health Science and Medicine, which was subsequently forwarded to the department heads. Participants in the study gave their written agreement and were notified of their right to refuse to participate. The information's confidentiality was protected via coding. The interview was conducted in a private space away from other people. The raw data obtained in the field was only accessible to authorized individuals.

## Results

**Sociodemographic characteristics of the respondents:** out of the totally 748 students, seven hundred thirty-five students participated in this study with the overall response rate of 98.2%. Majority of the study participants (61.2%) was males. Five hundred forty-four (74%) respondents were in the age group of 22-24 years and the rest were below or under the stated age group. With regard to their religion, majority (45.6%) of the respondents was orthodox followers followed by protestants which was found to be 201 (27.3%). Out of 450 male students, 341 (76%) consume alcohol with the rest 109 (24%) don´t consume. Out of 285 female students, 105 (37%) consume alcohol. One hundred fifty (74.6%) Orthodox religion followers were found to be alcohol consumer. Out of 543 chat chewers, 357(65.7%) consumes alcohol whereas 89 out of 192 non-chewers (46.4%) had been consuming alcohol. Out of 468 students who earn monthly pocket greater than 500ETB, 301 (64.3%) consume any kind of alcohol whereas 130 students out of 267 (48.6%) those getting less than 500ETB monthly pocket money were found to consume alcohol With regard to practical history of the families and peers of the respondents, 262 students from 314 family history of drinking alcohol (83.4%) consume any kind of alcohol (Table 1).

**Table 1:** socio-demographic characteristic of respondents in Wolaita Sodo University students, 2021

Variables	Category	Frequency	Percent
Sex	Male	450	61.2
	Female	285	38.8
Age	18-21	43	52
	22-24	33	61
	≥25	73	67
Religion	Protestant	201	27.3
	Orthodox	335	45.6
	Catholic	106	14.4
	Muslim	93	12.7
Occupation of HH	Gov employed	182	61
	Merchant	155	70
	Farmer	106	50
	Daily labor	2	40
	House wife	1	100
Monthly income (pocket money)	<500	130	49
	501-1000	215	64
	1001-1500	61	76
	1501-2000	25	81
	≥2001	15	75

**Figure 2:**
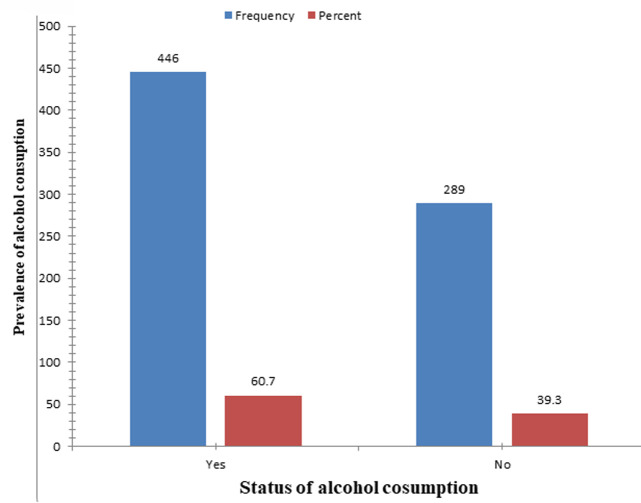
prevalence of alcohol consumption among undergraduate graduating students of Wolaita Sodo University, 2021

**The prevalence of alcohol consumption among undergraduate graduating from Wolaita Sodo University students:** the prevalence of alcohol consumption among undergraduate graduating students of Wolaita Sodo University was 60.7% (AOR= 0.02, 95% CI: 0.55-0.65) the majority of male consume 341 (46.4%) and female 105(14.3%) consume alcohol (Figure 2).

**Knowledge and attitude respondents towards on alcohol consumption:** regarding knowledge and attitudes of students on alcohol consumption and its effect, 387out of 735 (52.6%) students who knows well about the effect of alcohol consumption take alcohol whereas the rest 348 (47.4%) don´t know well about the effect of alcohol consumption and majority of students 429 (57.7%) of the respondents were aware that alcohol consumption can cause health and nutritional related chronic diseases such as liver cirrhosis, 278 (38%), type 2 diabetes mellitus, heart disease 37 (5.8%), 36 (4.8%) respectively. Whereas students who don´t know that alcohol consumption can cause liver cirrhosis 457 (62%), diabetes mellitus 698 (94%) heart disease 699 (95%) and students who know about alcohol can cause malnutrition 315 (42.3%) don´t know about alcohol can cause malnutrition 420 (56.5%) (Table 2). Majority of students 335 (45.6%) totally disagree that alcohol consumption as relaxing and 400 (54.4%) agree taking any kind of alcohol as relaxing mechanism and the respondents were 422 (57.4%) totally disagree that alcohol consumption as nutritious and 313 (42.5%) agree attractive any type of alcohol consumption as nutritious (Table 3).

**Table 2:** knowledge of respondents towards effects of alcohol consumption

Variables	Category	Frequency	Percent
Can alcohol affect health	Yes	387	52.6
	No	348	47.3
Alcohol important for health	Yes	307	41.7
	No	428	58.2
Alcohol causes health related diseases	Yes	429	58.3
	No	306	41
Types of health related diseases	Liver disease	278	37.8
	Diabetes	37	5.8
	Heart disease	36	4.8
Alcohol can cause malnutrition	Yes	315	42.3
	No	420	56.5

**Table 3:** attitudes towards alcohol consumption

Variables	Category	Frequency	Percent
I feel relaxed when I drink alcohol	Strongly disagree	93	12.6
	Disagree	191	26
	Agree	183	25
	Neutral	92	12.5
	Strongly agree	176	24
I drink alcohol to get care and appreciations from my colleagues	Strongly disagree	108	14.5
	Disagree	172	23
	Neutral	170	22.4
	Agree	178	23.9
	Strongly agree	107	14.4
I drink alcohol to get grace and appreciations from family and/or community	Strongly disagree	72	9.7
	Disagree	251	34
	Neutral	106	14.2
	Agree	206	27.7
	Strongly agree	100	13.4
Alcohol is nutritious	Strongly disagree	71	9.7
	Disagree	316	42.3
	Neutral	35	4.7
	Agree	240	32.6
	Strongly agree	73	10

Peer pressure factors: out of 432 students whose peers consume alcohol, 391 (90.5%) replied that they consume alcohol whereas 55 (18.2%) students out of 303 of those peers having no history of alcohol consumption only consume alcohol. Out of 543 chat chewers, 357 (65.7%) consumes alcohol whereas 89 out of 192 non-chewers (46.4%) had been consuming alcohol.

Environmental factor: regarding the availability of 65.2% of the students can easily avail alcohol to buy and consume nearby the campus compound can easily get alcohols of their interest without much effort of alcohol consumption among University students and 69.7% of the students who participated in the study can access alcohol in University students as these are for youths in school contribute to alcohol consumptions.

**Factors associated with alcohol consumption among undergraduate graduating students of Wolaita Sodo University:** bivariate logistic regression analysis was identified predictor variables alcohol consumption. During bivariate analyses, variables that were associated with alcohol consumption among undergraduate graduating students of Wolaita Sodo University where sex, alcohol consumption history of family and peers, cultural effect, personal history of chewing chat and smoking cigarettes, knowledge of individuals on effect of alcohol and their perception on consuming alcohol. However, during multi-variate analysis, sex, alcohol consumption history of family, drinker friends, being smoking cigarettes and knowledge on effect of alcohol were significantly associated with alcohol consumption. The current study showed that female students were (AOR=0.34, 95% CI: 0.21-0.54) less likely to consume alcohol when compared with males. Similarly, students who came from the society where drinking alcohol is not culturally forbidden were (AOR=0.41, 95% CI: 0.23-0.74) less likely to consume alcohol when compared with those who came from the society where drinking alcohol is culturally forbidden. This study also revealed that students whose family members consumes alcohol regularly were approximately five times (AOR= 4.83, 95% CI: 2.68-8.70) more likely to consume alcohol when compared with those students who had no history of familial alcohol consumption. Whereas, students whose peers (friends) had no practice of drinking alcohol were (AOR=0.03, 95% CI: 0.02-0.06) less likely to consume alcohol when compared with those students who had drunk friends. Those students who don´t chew ´´chat ‘´ were AOR=0.45 (.32-.63) less likely to consume than those students who chew chat. On the other hands, individual factors? Students who had no personal history of smoking cigarettes were (AOR= 0.49, 95% CI: 0.29-0.88) less likely to consume alcohol when compared with smokers. Knowledge about who students who don´t know well about the effect of consuming alcohol were around 2.7s times (AOR= 2.71, 95% CI:1.67-4.50) more likely to consume alcohol when compared with those having better knowledge on the issue (Table 4).

**Table 4:** factors associated with alcohol consumption among undergraduate graduating students of Wolaita Sodo University

Variable	Category	Consume alcohol		COR (95%CI)	AOR (95%CI)
		Yes	NO		
Sex	Male	341(76%)	109 (24%)	1	1
	Female	105 (37)	180 (63)	0.18(0.13-0.25)	0.34 (0.21-0.54)
Religion	Protestant	167	168	1	1
	Orthodox	150	51	2.96 (2.02-4.34)	0.95 (0.50-1.78)
	Catholic	77	29	2.67 (1.66-4.31)	0.50 (0.23-1.11)
	Muslim	52	41	1.28 (0.8-2.02)	0.96(0.43-2.01)
Chew chat	Yes	350	186	1	1
	No	89	103	2.21 (1.59-3.10)	0.45(0.32-0.63)
Smoking	Yes	377	208	1	1
	No	69	81	47(.33-.68)	0.49 (0.29-0.88)
Drunk family	Yes	262	52	6.49 (4.55-9.25)	4.83 (2.68-8.70)
	No	184	237	1	1
Drunk friends	Yes	391	41	1	1
	No	55	248	0.02 (0.02-0.04)	0.03 (0.02-0.06)
Culture effect	Yes	387	187	1	1
	No	59	102	0.28(0.19-0.40)	0.41(0.23-0.74)
know about effect of alcohol consumption	Yes	244	219	1	1
	No	202	70	2.59 (1.87-3.59)	2.71 (1.67-4.50)
Perceive alcohol consumption as relaxing	Agree	93	41	1.59 (1.07-2.38)	1.28 (.72-2.26)
	Disagree	353	248	1	1

## Discussion

The prevalence of alcohol consumption among Wolaita Sodo University undergraduate gradating regular students was 60.7% (AOR= 0.02, 95% CI: 0.55-0.65); the current study's findings were relatively high levels of alcohol consumption when compared to other university students surveys conducted in Jigjig (27.3%), Addis Ababa (31.4%), Gondar (48.23%), and Hawassa University (40.8%) [24-26]. In other words, this study found that the highest reported prevalence of alcohol consumption was (19.6% -35%) [27]; probable reasons for the disparity include geographical differences, differences in study times, and the availability of alcohol beverage within walking distance of campuses. However, the current study's alcohol consumption prevalence was significantly lower than similar studies conducted at Dilla University (67.6%), Debra Marko's University (79%), and the University of South Africa (70%), respectively [25,27,28]. The likely differences were due to socio demographic factors, study areas, source availability in the area, and study timing. According to multiple logistic regression, female students are 66% less likely than male students to consume alcohol (AOR= 0.34, 95% CI: 0.21-0.54), which is consistent with a WHO report [9]. Students whose family members consume alcohol on a regular basis were approximately 4.8 times (AOR= 4.83, 95% CI: 2.68-8.70) more likely to consume alcohol than those students who had no history of familial alcohol consumption. The findings were comparable to those of a study conducted at Dilla University, which found that having alcoholic family members was highly related with alcohol intake (AOR 1.29 95% CI:0.89-1.86) [25]. When compared to students who had drunker friends, those who did not have drunker friends were 97% (AOR=0.03, 95 percent CI: 0.02-0.06) less likely to consume alcohol, and the findings were similar to those of a study conducted in Debar Markos University students (AOR= 0.34, 95% CI: 0.21-0.54) [27]. This study found that there was a significant link between alcohol consumption and knowledge of the effects of alcohol, with students who didn't know much about the effects of drinking being 2.7 times (AOR= 2.71, 95% CI: 1.67-4.50) more likely to consume alcohol than those who knew more about the effects of drinking. Students who do not chew “chat” are 55% less likely to consume than those who do (AOR= 0.45, 93% (.32-.63)). According to the findings, chewing “chat” was significantly associated with alcohol consumption among students at Dilla University [25]. When compared to non-smokers, students who had no personal history of smoking cigarettes were (AOR= 0.49, 95% CI: 0.29-0.88) less likely to use alcohol. This finding matches that of research conducted at Debre Markos University [27].

Limitation: lack of biochemical measures to assess alcohol consumption deeply. The cross-sectional nature of the study design might not show the cause and effect relationships between alcohol consumption and other variables.

## Conclusion

When compared to prior studies on similar research populations and associated factors, the prevalence of alcohol consumption among undergraduate graduating students of Wolaita Sodo University was quite high. Alcohol consumption was associated to being female, family history of alcohol use, chew “chat”, smoking cigarette, drunker friends, and knowledge of the effects of alcohol. The responsible bodies (health office, university authorities, parents, education sector, and other stakeholders) should work together to adopt strategies for preventing, detecting, and improving alcohol consumption among students. Psychosocial counseling and sensitization therapy for students should be prioritized. The number of students who drink was reduced by establishing an anti-psychoactive substance club on campus, which was aimed to modify students' opinions about drinking.

### What is known about this topic

- *Alcohol consumption is a major public health problem issue in the worldwide;*
- *Alcohol consumption is an important risk factor for many socioeconomic and health problems in many societies which is especially affects young common among the University students*.

### What this study adds

- *The prevalence of alcohol consumption among undergraduate graduating students of Wolaita Sodo University was likely high;*
- *Sex, family history of alcohol use, chew “chat”, smoking cigarette, drunker friends, and knowledge of the effects of alcohol were significantly associated with alcohol consumption;*
- *The responsible bodies (health office, university authorities, parents, education sector, and other stakeholders) should work together to adopt strategies for preventing, detecting, and improving alcohol consumption among undergraduate graduating students of Wolaita Sodo University*.
